# A retrospective case series of eight patients with epidermal growth factor receptor inhibitor–induced cutaneous toxicities managed with dupilumab

**DOI:** 10.1016/j.jdcr.2024.08.031

**Published:** 2024-09-16

**Authors:** Fiorinda F. Muhaj, Jessica Tran, Julia Dai, Omar Pacha, Anisha B. Patel

**Affiliations:** a Department of Dermatology, University of Texas MD Anderson Cancer Center, Houston, Texas; b Department of Dermatology, University of Texas Health Science Center Houston, Houston, Texas

**Keywords:** acneiform eruption, cancer care, cutaneous toxicity, dermatitis, dupilumab, eczematous dermatitis, epidermal growth factor inhibitor, erosive pustular dermatosis, oncodermatologist, oncodermatology, pruritus, targeted therapy, toxicity

## Introduction

Epidermal growth factor receptor inhibitors (EGFRi) comprise small-molecule receptor tyrosine kinase inhibitors and monoclonal antibodies approved for several malignancies including breast, head and neck, pancreatic, colorectal, and lung cancer. In addition to being expressed on solid tumors, the epidermal growth factor receptor (EGFR) is present in the epidermis, its appendages, and dermal capillaries.^1^^,^^2^ Consequently, its inadvertent inhibition in the skin leads to a wide range of dermatologic adverse effects (AEs) that profoundly affect quality of life and chemotherapy adherence. Severe cutaneous AEs can lead to targeted therapy dose modification or discontinuation in 36% and 72% of the patients, respectively.^1^^,^^2^

Acneiform eruption develops within 1-2 weeks of EGFRi initiation and is the most frequent EGFRi-induced cutaneous toxicity. Depending on the agent, it affects 60% to 80% of the patients with a higher incidence for monoclonal antibodies.^1^, ^2^, ^3^, ^4^ All-grade acneiform eruption develops in 75% to 90% of the patients, while severe grade 3 or 4 rash can be seen in 10% to 20% of patients.^5^

A third of patients on an EGFRi develop eczema-like changes, including pruritus, progressive xerosis, and fissuring.^1^^,^^4^^,^^6^^,^^7^ A localized variant of eczematous dermatitis presents with palmoplantar deep fissuring and dryness. Xerosis and eczematous dermatitis often accompany or succeed the acneiform eruption, usually after 30-60 days of therapy.^5^

A less frequent yet significantly burdensome toxicity is erosive pustular dermatosis (EPD)-like eruption of the scalp, which characteristically presents with thick matted crusting, erosions, and pustules leading to follicular destruction and scarring alopecia.^8^

The severity of skin toxicity is often graded based on the National Cancer Institute Common Terminology Criteria for Adverse Events (NCI-CTCAE v5), which does not consider body location or the effect on quality of life, underestimating the true impact of EGFRi-induced cutaneous AEs.^5^ The Multinational Association of Supportive Care in Cancer (MASCC) has published treatment guidelines for acneiform eruptions. Grade 1 acneiform eruptions are managed with topical agents, including emollients, corticosteroids, clindamycin, or dapsone. Patients with grade 2 rash are managed with systemic antibiotics, more commonly doxycycline or minocycline. For patients with nonresponsive grade 2 or grade 3 acneiform eruption, a short course of systemic corticosteroids can be considered in addition to low-dose retinoids such as acitretin or isotretinoin.^2^^,^^4^ Notably, there are no therapeutic guidelines for EGFRi-induced eczema or EPD-like eruption.

As EGFRi-induced cutaneous toxicities tend to co-occur, management can be challenging. Additionally, the available armamentarium carries a lot of limitations. Systemic antibiotics are often required for prolonged courses of 2 m or longer and only demonstrate nondurable and modest improvement.^4^^,^^9^ On the other hand, systemic retinoids are poorly tolerated due to overlapping AEs with EGFRi, such as eczema, photosensitivity, dryness, cheilitis, or pyogenic granulomas, in addition to an increased risk of superinfection.^4^ Similarly, the impact of systemic corticosteroids in patients’ survival and cancer outcome remains controversial.^10^

There is an unmet need for therapies in patients with severe eruptions refractory or intolerant to the above agents.

In this case series, we present the characteristics and outcomes of patients with refractory EGFRi-induced cutaneous toxicities managed with dupilumab.

## Methods

### Patients

Patients at MD Anderson Cancer Center (MDACC) in Houston, TX, referred to our oncodermatology service between January 1, 2020, and October 23, 2023, for EGFRi-related cutaneous toxicity and who received a prescription for dupilumab were identified using our electronic medical record (*n* = 26). Patients’ electronic health records were retrospectively reviewed by the authors. Patients were excluded from our series if their cutaneous toxicity was not attributed to an EGFRi (*n* = 8), if they were not evaluated by our oncodermatology service (*n* = 5), if dupilumab was prescribed but not started (*n* = 4), or if the patients were not evaluated in dermatology post-dupilumab (*n* = 1). Patients were eligible for inclusion if they were on systemic therapy with an EGFRi, were prescribed dupilumab for refractory EGFRi-induced eczematous dermatitis, had another concurrent EGFRi-induced cutaneous toxicity, and were evaluated after starting dupilumab (*n* = 8).

Data collected included patient characteristics such as age, sex, primary cancer type and stage, and current and prior cancer therapy exposures. Information regarding their cutaneous toxicities, timing and management, treatment response, therapy alteration, and overall patient outcome were gathered. Medical photographs and CTCAE grade of cutaneous toxicities were not available or could not be inferred for all patients from the retrospective chart review. Qualitative descriptors were used to assess treatment response.

### Therapy

All patients received dupilumab subcutaneously (SC) based on atopic dermatitis dosing, 600 mg SC initially followed by 300 mg SC every 14 days. Coverage for dupilumab was obtained through patients’ insurance as standard of care for severe and refractory atopic dermatitis. All patients continued topical corticosteroids and some patients received other systemic therapy while on dupilumab. Assessments such as “clear, resolved, markedly improved, or improved” were defined as responses to dupilumab. Nonresponse was defined by documented assessments such as “persistent, worsening, failing treatment, or not improving.”

## Results

### Patient characteristics

We identified 8 patients, median age of 65 years (range, 55-88) at the time of presentation to dermatology, who were on therapy with an EGFRi for clinical stage IV metastatic cancer. Patient demographics and toxicity characteristics are summarized in Tables I and II.

**Table I:** Patient demographics, oncologic, and toxicity characteristics

Total number of patients (*n*)	8
Patient age (median, range)	(65, 55-88)
Patient race (*n*)	
White	6
Hispanic or Latino	1
Asian	1
Sex	
Female (*n*)	6
Male (*n*)	2
EGFRi-induced cutaneous toxicity (*n*)	
Eczematous dermatitis	8 of 8
Pruritus	8 of 8
Acneiform eruption	7 of 8
Paronychia	5 of 8
EPD-like eruption	3 of 8
Systemic therapies prior to dupilumab	
Tetracycline	
Doxycycline	6 of 8
Minocycline∗	2 of 8
Corticosteroids	6 of 8
Retinoid	
Acitretin∗	4 of 8
Isotretinoin∗	2 of 8
Dapsone†	3 of 8
Average duration of systemic therapies	Average duration, range
Retinoid	
Isotretinoin	5.8 (0.5-11) mo
Acitretin	4.6 (0.5-12) mo
Dapsone	6.3 (2-0) mo
Tetracycline	
Doxycycline	2.6 (1-3) mo
Minocycline	1.8 (0.5-3) mo
Corticosteroids	31 (6-45) d
Time from EGFRi initiation to first documentation of skin toxicity	Average duration, range
Eczematous dermatitis	3.6 (1-9) mo
Acneiform eruption	5.6 (0.6-18) wk‡
EPD-like eruption	3 (1-4) mo
Time from toxicity documentation to first dupilumab administration	Average duration, range (mo)
Eczematous dermatitis	9.3 (1-14)
Acneiform eruption	6.6 (1-19)
EPD-like eruption	2.8 (0.25-7)
*Staphylococcus aureus* superinfection (*n*)§	
Acneiform eruption (overall)	5 of 7
Before dupilumab	5 of 5
After dupilumab	1 of 5
EPD-like eruption (overall)	2 of 3
Before dupilumab	-
After dupilumab	2 of 2

*EGFRi*, Epidermal growth factor receptor inhibitor; *EPD-like eruption*, erosive pustular dermatosis-like eruption of scalp.

∗ Three patients with acneiform eruption were continued on minocycline, acitretin, and isotretinoin while on dupilumab.

† Two patients with EPD-like eruption continued dapsone while on dupilumab.

‡ One patient was excluded in this calculation due to inability to infer time onset from documentation.

§ Superinfection was culture proven and positive for methicillin-sensitive *S aureus* and methicillin-resistant *S aureus* in 1 patient with acneiform and 1 patient with EPD-like eruption.

**Table II:** Characteristics of EGFRi-induced cutaneous toxicity and response to dupilumab

*n*	Malignancy	EGFRi (+ other cancer therapy)	Mechanism of action	Acneiform eruption distribution	Systemic medications before dupilumab	Concurrent systemic therapies with dupilumab	Acneiform eruption response to dupilumab	Eczematous dermatitis distribution	Eczematous dermatitis response to dupilumab	EPD-like eruption response to dupilumab	Changes to EGFRi therapy while on dupilumab	Previous EGFRi exposure
1	Lung adenocarcinoma	Osimertinib	TKI to EGFR mutations (T790 M, L858 R, and exon 19 deletions)	Scalp	Corticosteroid taper	None	Marked improvement followed by complete clearance	Hands	Improved but persistent	n/a	None	-
2	Lung adenocarcinoma	Poziotinib	TKI to EGFR exon 20 mutations	Face, trunk, extremities	Doxycycline Acitretin Corticosteroid taper	Acitretin	Marked improvement	Hands, trunk, extremities	Marked improvement of diffuse eczema and resolution of hand dermatitis	n/a	Dose reductions Drug holidays Ultimate discontinuation due to PD along with GI and skin toxicity	Afatinib Osimertinib
3	Lung adenocarcinoma	Amivantamb + osimertinib	Bispecific EGF + MET receptor mAb/TKI	Trunk, extremities	Doxycycline Corticosteroid taper	Prednisone taper	Improvement	Trunk, extremities	Improvement	n/a	Amivantamab drug holiday Both agents discontinued due to no response	Osimertinib monotherapy
4	Lung adenocarcinoma	Amivantamab + osimertinib	Bispecific EGF + MET receptor mAb/TKI	n/a	Doxycycline Dapsone	Dapsone 50 mg every other day due to hemoglobin intolerance Colchicine	n/a	Trunk, extremities	Improved and well controlled	Persistent requiring addition of colchicine	Two amivantamab dose reductions and one treatment holiday	-
5	Head and neck squamous cell carcinoma	Cetuximab	EGFR mAb	Scalp, face, trunk, extremities	Doxycycline Acitretin Isotretinoin Minocycline Corticosteroid taper	Minocycline	Marked improvement	Scalp, face, trunk, extremities	Marked improvement	n/a	Discontinued due to PD	-
6	Colorectal adenocarcinoma	Investigational agent	EGFR/MET bispecific antibody	Face, trunk, extremities	Doxycycline Acitretin	Prednisone taper Dapsone	Initial improved with breakthrough flares	Hands	Persistent requiring addition of narrow band UVB	Persistent	Dose reduction and drug holiday due to skin toxicity. Drug discontinued due to PD	Cetuximab
7	Anal squamous cell carcinoma	Investigational agent + pembrolizumab	TGF beta/EGFR bispecific antibody + PD-1 mAb	Face, trunk, extremities	Doxycycline Isotretinoin Minocycline Dapsone	Low-dose isotretinoin	Initial improvement with breakthrough flares managed by adding low-dose isotretinoin with great control	Face, extremities	Marked improvement	Resolved prior to dupilumab	Drug holiday and systemic prednisone due to non-skin related toxicities	-
8	Anal squamous cell carcinoma	Investigational agent + pembrolizumab	TGF beta/EGFR bispecific antibody + PD-1 mAb	Face, trunk	Doxycycline Acitretin	Narrow band UVB therapy∗	Acneiform eruption resolved prior to dupilumab	Face, trunk, extremities	Marked improvement	n/a	Prednisone taper and treatment hold due to lichenoid dermatitis attributed to pembrolizumab	-

*EGF*, Epidermal growth factor; *EGFR*, epidermal growth factor receptor; *EGFRi*, Epidermal growth factor receptor inhibitor; *EPD-like eruption*, erosive pustular dermatosis-like eruption of scalp; *GI*, gastrointestinal; *mAb*, monoclonal antibody; *MET*, mesenchymal-epithelial transition; *PD*, progression of disease; *PD-1*, programmed cell death protein 1; *TGF*, transforming growth factor; *TKI*, tyrosine kinase inhibitor.

∗ Phototherapy was started due to pembrolizumab-induced dermatitis.

All (8 of 8) patients had EGFRi-induced eczematous dermatitis. Twenty-five percent (2 of 8) of patients had localized eczema of the hands, while the remainder had generalized eczematous dermatitis. All patients with EGFRi-induced eczema had failed topical corticosteroids and oral antipruritics necessitating therapy escalation to dupilumab.

Eighty-eight percent (7 of 8) of the patients had concurrent acneiform eruption, 86% (6 of 7) of whom received an oral tetracycline. One patient had a history of doxycycline anaphylaxis and thus was not a candidate for tetracycline therapy. All patients with acneiform eruption that were candidates for tetracycline therapy (6 of 6) demonstrated no improvement on doxycycline. Subsequently, 33% (2 of 6) of patients were transitioned to oral minocycline with no response. Seventy-one percent (5 of 7) of patients with acneiform eruption received a systemic retinoid, including acitretin (4 of 7) and isotretinoin (2 of 7). One patient was transitioned from acitretin to isotretinoin due to breakthrough flares with no significant improvement. The average number of nonsteroidal systemic therapies for acneiform eruption before dupilumab was 2.7 over an average of 6.6 m.

Thirty-eight percent (3 of 8) of patients in our cohort developed EPD-like eruption. In addition to localized therapy with topical corticosteroids and systemic tetracyclines or retinoids for underlying acneiform eruption, all patients with EPD-like eruption of the scalp (3 of 3) were started on dapsone. Patients with EPD-like scalp eruption received on average 2.7 different nonsteroidal systemic therapies prior to dupilumab initiation.

### Response to dupilumab

The patients were followed for an average of 5.4 m (range, 1-11 m) post-dupilumab initiation. Among patients with eczematous dermatitis, 75% (6 of 8) demonstrated sustained response to dupilumab therapy, while 25% (2 of 8) had persistent symptoms. Response was noted after an average of 4.5 doses of dupilumab, including the loading dose. Interestingly, both patients with persistent eczema post-dupilumab had localized hand dermatitis requiring the addition of ruxolitinib 1% cream.

Among patients with acneiform eruption, dupilumab response was defined clinically as resolution, improvement, or marked improvement of pustules, papules, and erythema. One patient had acneiform eruption resolution prior to initiation of dupilumab and remained rash free while on therapy. Of the remaining 6 patients with active acneiform rash at the time of dupilumab administration, 67% (4 of 6) demonstrated sustained improvement or sustained marked improvement through the follow-up period. Thirty-three percent (2 of 6) of the patients achieved initial improvement followed by breakthrough flares that were managed with low-dose isotretinoin in 1 patient and the addition of dapsone in the other patient. The initial response in acneiform eruption was noted after an average of 3.1 (range, 2-8) doses of dupilumab, including the loading dose.

Among the 3 patients with EPD-like eruption, one experienced resolution of scalp eruption prior to dupilumab while on EGFRi therapy. The remaining 2 patients experienced no improvement in the clinical findings or symptoms post dupilumab initiation.

Topical corticosteroids were continued on all patients while on dupilumab therapy. Systemic corticosteroids for skin toxicity were administered to 63% (5 of 8) of patients, 4 patients with acneiform eruption, and 1 patient with EPD-like eruption. Two patients with acneiform eruption were on a systemic corticosteroid taper at the time of dupilumab initiation. Among the 2 patients with EPD-like scalp eruption patients, nonsteroidal systemic therapies continued concurrently with dupilumab included dapsone in both and colchicine in 1 patient due to continued flares. Three patients received nonsteroidal systemic therapies for acneiform eruption while on dupilumab including acitretin, isotretinoin, and minocycline, each. In patients with EGFRi-induced eczematous dermatitis, 1 patient with hand dermatitis and 1 patient with generalized eczema received narrow band UVB therapy while on dupilumab.

### Changes to EGFRi and cancer outcome

During the overall course of treatment, the severity of skin toxicity led to EGFRi drug holiday in 38% (3 of 8) of patients and therapy discontinuation in 25% (2 of 8) of patients. At the end of this review, 63% (5 of 8) of patients had achieved stable or stable to improved metastatic disease and 13% (1 of 8) of patients had disease progression. Twenty-five percent (2 of 8) of patients died due to metastatic cancer, both 5 years after cancer diagnosis.

## Discussion

The development of EGFRi-induced cutaneous toxicity, specifically acneiform eruption, is a positive predictor of overall response rate and survival.^3^^,^^4^^,^^6^ Although less robust, there are data to suggest increased treatment response with rash severity. These correlations hold true across different tumors including head and neck, lung, and colorectal cancer.^5^^,^^11^ Consequently, the appropriate management of EGFRi-induced skin toxicities is critical to maximize the duration of therapy and antitumor response.

The exact pathophysiology of EGFRi-induced cutaneous toxicities is not well characterized. EGFR inhibition in the skin leads to hyperkeratinization and abnormal desquamation, resulting in follicular plugging.^4^ Some evidence demonstrates that EGFRi interact with *Corynebacterium acnes* to upregulate interleukin (IL)-36 gamma and IL-8 mediating neutrophil chemotaxis in keratinocytes, explaining some of the mechanisms underlying acneiform eruptions.^6^ Additionally, EGFR blockade disrupts epidermal homeostasis leading to epidermal thinning, increased permeability, and increased inflammatory cytokines that recruit dendritic cells, macrophages, granulocytes, mast cells, and T cells.^1^^,^^12^ This is accompanied by decreased production of antimicrobial peptides and skin barrier proteins contributing to dryness, pruritus, and superinfections in a third of patients.^1^^,^^6^^,^^11^^,^^12^

These eczema-like changes succeed or co-occur with acneiform eruption suggesting at least some mechanistic link. Interestingly, elevated IL-4, a key T-helper 2 (Th2) cytokine implicated in intrinsic atopic dermatitis, has been found in non-lesional skin in EGFRi responsive patients who developed an acneiform eruption.^11^

Dupilumab is an IgG4 (IL)-4 receptor monoclonal antibody leading to targeted downregulation of the Th2 inflammatory axis. Approved dermatologic indications include moderate-to-severe atopic dermatitis and prurigo nodularis. Due to its targeted mechanism of action, it is also used off-label in the management of immunotherapy-related toxicities including pruritus, bullous pemphigoid, and eczematous dermatitis. Its role in the management of EGFRi skin toxicities is yet to be determined. However, from our case series, we can postulate that downregulation of the Th2 inflammatory signal in EGFRi-induced acneiform eruption and eczematous dermatitis can lead to clinical improvement.

Further studies are needed to elucidate the exact pathophysiology of EGFRi-induced skin toxicities to identify targeted, therapeutically actionable inflammatory markers. New agents are being developed that target EGFR or other proteins in the mitogen-activated protein kinase pathway. Consequently, we are expected to encounter a rise in the incidence and severity of EGFRi-induced skin toxicities highlighting the need for better therapeutic options. Our case series suggests that Th2 inflammation may be implicated in EGFRi-induced eczematous dermatitis and acneiform eruption. While there are no published data on the effectiveness of dupilumab for acneiform eruption without eczematous dermatitis, the utility of dupilumab in this setting deserves further investigation.

## Conclusion

Our findings are limited by the small sample size, lack of a control group, lack of a standardized assessment tool, and retrospective analysis. Additionally, all patients received concurrent topical or systemic agents while on dupilumab. However, we do not believe this to be a significant confounding factor since all patients on systemic monotherapy with retinoids, dapsone, or tetracyclines had an inadequate response until the addition of dupilumab. Though our patient sample is small, the response rates are clinically significant. They demonstrate dupilumab to be a promising and well-tolerated steroid-sparing alternative in cancer patients with EGFRi-induced acneiform eruption and eczema. Larger scale randomized controlled trials are warranted to support our findings.

## Conflicts of interest

None disclosed.
